# Assessing the Health Impact of the following Measures in Schools in Maradi (Niger): Construction of Latrines, Clean Water Supply, Establishment of Hand Washing Stations, and Health Education

**DOI:** 10.1155/2014/190451

**Published:** 2014-01-19

**Authors:** Halima Boubacar Maïnassara, Zilahatou Tohon

**Affiliations:** ^1^Centre de Recherche Médicale et Sanitaire (CERMES), 634 Boulevard de la Nation YN034, P.O. Box 10887, Niamey, Niger; ^2^College of Public Health, University of Kentucky, 111 Washington Avenue, Lexington, KY 40536-0003, USA

## Abstract

*Objective.* To assess the effect on health of the following measures in schools in Maradi (Niger): clean water supply, construction of latrines, establishment of hand washing stations, and health education. *Methodology.* It was a “before and after” intervention study on a sample of school children aged 7 to 12 years in the Maradi region. The interventions included building of latrines, supplying clean water, setting up hand washing stations, and teaching health education lessons. An individual questionnaire, analysis of stool samples, and a group questionnaire were administered to children and teachers, respectively. The threshold for significance was set at *P* < 0.05. *Results*. A statistically significant reduction in cases of diarrhoea and abdominal pains was noted after the project. Overall, carriage of at least one parasite increased from 7.5% before the project to 10.2% after it (*P* = 0.04). In the programme group schools, there was a statistically significant increase in the prevalence of *Hymenolepis nana*, from 0 to 1.9 (*P* = 0.02). Pinworm prevalence remained stable in this group but increased significantly in the control group. *Conclusions.* Putting health infrastructure in place in schools obviously had an impact on hygiene-related habits in the beneficiary schools and communities.

## 1. Introduction

Children in developing countries bear a heavy burden of respiratory tract diseases and diarrhoeal diseases. These diseases can however be prevented with some basic hygiene measures. Hand washing with soap is one of the principal means of preventing transmission of certain diseases [1]. It is generally acknowledged that using latrines can also interrupt the transmission of diarrhoeal disease [2]. Measures such as hand washing with soap, water supply, construction of latrines, and promoting general hygiene provide opportunities for improving children's health [3].

In 2006, six major French actors on the international solidarity scene came together to form an innovative public-private partnership, the alliance for development. Members of the alliance include institutions from both the public sector (Ministry of Foreign Affairs; Ministry of Economy, Finance, and Industry; and *Agence Française de Développement*) and the private sector (*Sanofi-Aventis*, *Institut Pasteur*, and *Veolia Environnement*). The aim is to take advantage of their complementary competences and networks in undertaking concrete action to contribute to improving local environmental and health conditions in developing countries. In order to have a positive impact on prevention of communicable diseases and thus on the health of the population in the long term, individual hygiene measures must be supported by environmental sanitation. The *Agence Française de Développement* (AFD) was the lead partner for the alliance's project in Niger. The agency is in charge of ensuring the smooth running of the project on water, environment, and health in schools, known as ESAMIS. *Veolia Environnement* is the lead agency for the private partners. The Maradi region of Niger was identified as the main intervention area for this pilot phase. The goal of the ESAMIS project is to improve access to clean drinking water, sanitation, and health education for school children and their teachers. The project focused on the following hygiene measures: water supply, with the construction of hydraulic infrastructure; sanitation, with the construction of latrines; proper management of this equipment through the education programmes and rules of hygiene. The ESAMIS project lasted 18 months, from January 2007 to June 2008 (Table 1). It was considered a success, since it met its objectives. The latrines were built, clean water was provided, school children in Maradi schools were educated on health and hygiene, and local management committees (comprising teachers and parents) were set up to ensure sustained use of the infrastructure.

There has been very little research on the hygiene and living conditions of communities in Niger and the positive effects that interventions produce for beneficiary populations. And yet it is important to assess how promoting hygiene impacts health [4].

The *Centre de Recherche Médicale et Sanitaire* (CERMES) was therefore requested to evaluate the epidemiological impact of the ESAMIS project. School children's performance is affected by the absence of hygiene and water. It is expected that putting in place hygiene and sanitation infrastructure and providing health education will lead to a reduction in symptoms of infection, absenteeism, and intestinal parasitic diseases carriage. Most of the existing research on the health impact of clean water supply and hygiene infrastructure projects focuses on the incidence of diarrhoeal diseases, malnutrition, and infant mortality [5–9]. Very little research has been carried out on the impact of hygiene and sanitation measures on intestinal parasitic diseases in sub-Saharan Africa. A study was carried out in Côte d'Ivoire to assess the impact of latrine construction, water supply, and health education on the incidence of parasitic diseases in children aged 2 to 4 years [10]. It is generally accepted in theory that latrines do serve to reduce the incidence of parasitic disease [11]. Also, when family heads are asked about household health problems and health problems in the community in general, they rarely ever mention intestinal worms or schistosomiasis as a major health problem [12]. It is therefore clear that more studies must be undertaken to document the epidemiology of parasitic infections and estimate their impact on the affected populations.

This paper is a report of the findings of the CERMES study. It is meant to consolidate the existing documentation on the impact of improved hygiene on intestinal parasitic diseases in sub-Saharan Africa. The aim is to assess how clean water supply, construction of latrines, establishment of hand washing stations, and health and hygiene education affected the health of school children in the Maradi region.

## 2. Methodology

### 2.1. Type of Study

This was a “before and after” intervention study on a representative sample of school children from schools in the Maradi region. The study was repeated 6 months later. It was also a “here and elsewhere” study since some schools were beneficiaries of the project (programme group) while others were not (control group).

### 2.2. Population Covered by the Study

The population covered by the study was made up of school children from 20 schools in the Aguie, Mayahi, and Tessaoua districts of the Maradi region (eastern Niger). The children were aged between 7 and 12 years.

### 2.3. Sample Selection

A double-stage sampling process was used.

Three primary schools (programme group) were first selected randomly, out of the 20 schools. For each school selected, another nonbeneficiary school (control group) was randomly selected from among all the schools within a 5 km radius of the programme group school.

120 pupils were then selected randomly from each of the 6 schools. For the two surveys, before and after the project, the same sampling plan was used for school children in both the programme group and the control group. The number of subjects needed to compare parasite carriage in the two groups of schools and demonstrate any difference in the programme group before and after the project was 720 in the six schools, that is, 120 per school.

The final sample was made up of school boys and girls aged between 7 and 12 years from public primary schools in the Awache and Magaria villages in the Tessaoua district, Bougouzawa and El Gueza in the Aguie district, and Zongon Oumara and Bassare in the Mayahi district.

### 2.4. ESAMIS Project Activities

Before the start of the project, the hygiene education programme for children in both groups was the standard government curriculum. This curriculum includes annual classes on hygiene and transmission of infectious diseases. Only two schools (one from each group) were equipped with two old latrines each. There were no clean water outlets in any of the schools.

The project set up clean water outlets, three latrines, and three hand washing stations in each of the “programme group” schools. This was followed by hygiene lessons. The assigned trainer in each of the programme schools held 30-minute daily discussion sessions with the school children and teachers. Representatives of parents and members of the school management committees also attended these sessions. The lessons focused on drinking water hygiene, use of latrines, and hand washing with soap. Four posters describing the use of latrines and hand washing techniques were distributed to each class. Teachers were given a package containing a guide describing the 5 steps in hand washing and including some basic concepts on the transmission of infectious diseases. The project provided drinking water in covered buckets equipped with a cup for each classroom in the programme schools. The school children were responsible for washing the equipment and changing the water each day. Teachers and the trainer taught the pupils how to use the latrine pits. Two plastic kettles were placed in each latrine for washing the anus. For hand washing, a cake of soap was placed on each washing bowl and the trainers demonstrated correct hand washing methods. This learning programme was based on the theory that these measures would inculcate the habit of hand washing with soap and using latrines. They are therefore expected to reduce the symptoms of infection, as well as children's absence from school. The training phase lasted 3 weeks. The teachers then continued to reinforce the educational message in the school programmes by repeating what they had learned in the training phase and by using the guide. Each week, the teachers would do a 10- to 15- minute revision of the messages in the posters on the wall. This entailed a quick review of the hygiene rules and the symptoms caused by lack of hygiene. This was done throughout the school year.

### 2.5. Practical Implementation of the CERMES Evaluation Study

The first field survey was carried out between November 7, 2007, and November 19, 2007, prior to the start of the programme, and the second between 20 and May 31, 2008.

#### 2.5.1. Data Gathering

An individual questionnaire was administered to all the school children to gather demographic data; information about symptoms such as diarrhoea, abdominal pains, and vomiting; water consumption habits; sources of drinking water at school; and latrine usage. The interviews were carried out in standard format in individual conversations between the school children and four trained interviewers who were well aware of how to conduct the interview, as well as the possible biases that could occur.

The teachers had to answer a group questionnaire to provide data on pupils' absence from school during the 5 days prior to the survey, as well as on the source, storage, and replenishment of drinking water at school. Teachers gave us a class by class list of pupils who had been absent.

Stool samples were collected from all the selected students. From these specimens two Kato-Katz slides were prepared and examined according to a previously described method [13, 14]. The aim was to find the eggs of *Schistosoma mansoni*, hookworm, pinworms, and other parasites (*Entamoeba coli*, *Entamoeba histolytica*, *Taenia saginata*, *Hymenolepis nana*, and *Trichomonas intestinalis*). The same collection methods were used during both CERMES surveys.

### 2.6. Data Management and Analysis

Data were entered into the database using Epi Info (CDC, Atlanta, GA, USA). The data was then analysed with the SPSS 11.5 software. The proportions were then compared using Pearson's chi-squared test and Fisher's exact test. Means were compared using Student's *t*-test. The threshold for significance was set at *P* < 0.05.

The factors studied were intestinal parasitic disease, symptoms caused by lack of hygiene, water consumption habits, absence from school, sources of clean drinking water at school, and latrine use. The main evaluation criterion was prevalence of intestinal parasites.

### 2.7. Ethics

The study was approved by the national ethics committee of Niger before the data were collected.

*Consent.* Only children whose parents had given their approval were allowed to participate in the study. Parents had first been briefed on the study goals and methodology. The primary school inspectorates of the schools concerned were also informed about the study, for each of the CERMES studies. The school director was first briefed and subsequently accompanied a delegation including members of the mission to meet the village chief and inform him of the aims of the study. Information was then passed on to parents through the parents' association, of which most of them were members.

*Advantages for Participants.* Children who were infected by intestinal helminths were given the appropriate treatment by the study team. For *Schistosoma mansoni* infection they were treated with praziquantel, and with albendazole for the other helminths.

## 3. Results

### 3.1. Data from the Questionnaire

#### 3.1.1. Sex and Age of Pupils

Two samples of children aged between 7 and 12 years were selected. During the preproject study, 665 pupils were selected, with a predominance of boys (sex ratio = 1.8). For the postproject study, 696 pupils were selected. 36.9% of them were girls (Table 2). The average ages of the children were 9.1 and 9 years, respectively, before and after the project.

#### 3.1.2. Functional Signs due to Lack of Hygiene

A statistically significant reduction in cases of diarrhoea and abdominal pains was noted after the project; from 3.9% before to 2.7% after, *P* = 0.04, and from 4.7% to 3.2%, *P* = 0.02, respectively (Table 2). This reduction was noted in both the programme group schools and those in the control group. There was no statistically significant difference in the prevalence of vomiting before and after the project (from 1.7% to 1.3%, *P* = 0.08).

#### 3.1.3. Absence from School

The number of children absent from school in the 5 days preceding the study increased significantly from 2.6% to 7.3% (*P* < 0.001). The increase in absence from school was statistically significant in both the programme group schools and those in the control group. The increase was however greater in the control group schools. The rate went up from 4.4% before the project to 8.6% (*P* = 0.01) after the intervention in the schools in the programme group and from 0.6% to 25.4% (*P* < 0.0001) in schools in the control group (Table 2).

#### 3.1.4. Pupils' Water Consumption Habits and Sources of Drinking Water at School

According to 19.7% of respondents, the water consumed in schools on the eve of the study came mostly from the water tap (61.1%). In schools in the programme group, consumption of tap water increased from 87.0% to 99.6% (*P* < 0.001), to the detriment of the two other sources of supply.

Use of the water tap was clearly much higher in the programme group schools (87.0%). In schools in the control group, water mainly came from the well (89.7% of the 12.1% respondents). Use of the borehole was significantly higher in schools in the control group, from 10.3% to 42.1%, *P* < 0.001 (Table 3). Following the project activities, schools in the programme group totally abandoned the use of the well as a source of water supply (from 10.9% to 0%).

Water was stored in buckets that were washed every two to seven days.

#### 3.1.5. Latrine Use at Home

There was no difference in the frequency of latrine usage at home in the programme group: from 24.8% to 29.8%, *P* = 0.20. This frequency however fell significantly in schools in the control group, from 6.8% to 3.3% (*P* = 0.01) Table 2.

### 3.2. Parasitology Findings

At the time that stool samples were taken for the preproject study, 7.5% of the 664 school children who provided such samples were carriers of at least one intestinal parasite. Prior to the intervention, the intestinal parasites carriage rate was significantly higher for the programme group schools than for those in the control group (9.6% versus 5.3%, *P* = 0.02). Among those who had worms, 62% were not using latrines (*P* = 0.004).

Overall, carriage of at least one parasite increased from 7.5% before the project to 10.2% after (*P* = 0.04) Table 4. This increase was influenced by the control group. It went from 9.6% before the project to 8.1% after the project in the programme group, although this was not statistically significant (*P* = 0.24). There was however a significant increase in the control group, from 5.3% before the project to 12.5% after it (*P* < 0.001).

After the project, parasite carriage was higher in the control group schools, compared to the programme schools, although the results barely reached the significance threshold (8.1% versus 12.5%, *P* = 0.05). Only carriage of parasites other than pinworm, *Ancylostoma,* and *Ascaris* was significantly higher in the control schools (*P* = 0.03).

No *Schistosoma mansoni* or *Taenia saginata* infections were found. Prevalence of the other intestinal parasites was lower. Details can be found in Table 5. In the programme group schools, there was a statistically significant increase in the prevalence of *Hymenolepis nana*, from 0 to 1.9 (*P* = 0.02). Pinworm prevalence remained stable in this group but increased significantly in the control group.

## 4. Discussion

This is the first ever study in Niger to describe the health impact of sanitation and hygiene for school children. A similar study has been done in Côte d'Ivoire [10].

It demonstrated a significant increase in the use of tap water systems as a source of water supply and in the adoption of hygiene measures. Intestinal parasitic diseases remained stable in the programme group but increased significantly in schools in the control group. This shows that construction of latrines, followed by a programme of health education, produces the required effects.

The frequency of diarrhoea and abdominal pains went down significantly. There was however no statistically significant reduction in cases of vomiting following the project. Vomiting is not a pathognomonic sign for lack of hygiene and may occur in other infections such as malaria. It is worth noting that the study took place in May, at the start of the high malaria transmission period. Under such conditions, and in the absence of a differential diagnosis, it is difficult to observe the real positive effects of latrines and water supply systems on the occurrence of vomiting.

The number of children absent from school in the five days preceding the survey increased in both the control schools and those in the programme group. There were however more children absent in the control group schools. A Chinese study covering a period of 5 months showed that children who had been taught about hand washing with soap missed school significantly less frequently that those who had not had such lessons [15]. It is of course difficult to link these observations directly with the project because the reasons for the children's absence are not always indicated. There may be several different reasons: truancy, farm work, and social events (births, weddings, deaths, etc.). In this specific situation, the principal reason was farm work. Indeed, the teachers often told us that the pupils had left the village in the company of their parents. May marks the start of the rainy season. The schools in the study are located in rural areas where families travel from the village with their children at this time, to work on their farms.

There was also an increase in reported use of the water supply system, latrines, and hand washing after using the toilet in all the schools, after the health education campaign. These complementary hygiene practices are known to be effective and they must be promoted in school. Pupils in the programme group schools made more frequent use of drinking water from the tap. The school drinking water was stored in buckets that were washed every two to seven days, depending on the school. The longer period between washes can be a source of contamination. Nevertheless, this method is still safer than the wells, which are sometimes not covered.

This study showed a significant increase in reported hand washing in schools in the programme group and to a lesser degree in schools in the control group. Reported latrine usage at home also increased slightly among pupils in the programme group schools. On the other hand, there was a reduction among children in the control group schools. This is almost certainly influenced by the hygiene education programme. The fact that children had been taught about promoting hygiene had a positive effect on the use of latrines at home [9]. These results may be due to the fact that very little time had elapsed between the project and the evaluation. Some of the reasons that may explain the low impact of hygiene interventions on the population include the fact that most studies last only for a short time and that interventions are not flexible enough in the way they are set up to include research methodologies [3]. Hand washing after using the toilet also increased in the programme group schools, which goes to show that the awareness-raising had been effective. The campaign, which was carried out in the schools, also focused on some material aspects such as providing water and availability of soap and latrines. Availability of soap close to the latrines and the time required to reach the water outlet are two of the constraints on the practice of hand washing after defecation [16]. In order to encourage people to wash their hands, constraining factors such as an appropriate source of water and soap availability have to be resolved [17].

Our study did not find any significant difference in parasite carriage prevalence in the programme group school. This prevalence however increased significantly in the control group schools. It was therefore not possible to demonstrate a significant reduction in parasitic diseases prevalence. In this, the study differed from what is sometimes reported in the literature [18]. It is however important to note that unlike the case of the control group, there was no increase in the prevalence of parasitic diseases in the programme group. The study carried out in Côte d'Ivoire demonstrated that improvements in hygiene conditions reduced the incidence of ascariasis by 75% and ancylostomiasis by 82%. The population included in that study was however younger than ours (2 to 4 years), and the study was carried out two to four years after the initial preintervention study [10].

In our study, overall carriage of intestinal parasites was low (7.5%) even before the project was implemented. Intestinal parasitic diseases are generally quite rare in Niger, with prevalence rates below 10% [19]. This low prevalence rate is also found in Chad and Mali, where the climate is similar to that of Niger [20]. The low prevalence of parasite carriage may also be due to the fact that deworming exercises using albendazole are carried out regularly in the Maradi region as part of school health programmes and also as part of neglected tropical diseases control activities [21]. Indeed a mass treatment campaign had taken place in the region in April 2008 and had covered both the schools in the programme group and those in the control group. For such a study, this represents a potential confounding factor that it was difficult to take into account.

The programme schools and the control group schools were close to each other (within a radius of 5 km). Children from the control group may therefore have heard about the project, and, as such, its real effects may actually be underestimated. In the duration of the study, children from the programme group may have disseminated the educational messages about hand washing, especially after defecation. Because of the methodological limitations, we were not able to assess which external factors may have influenced the hygiene practices of pupils (especially latrine usage).

Pupils' self-reported hand washing is not strongly correlated to observed behaviour [22], which requires immense resources [23]. As a result we did not assess changes in hand washing.

Although we were not able to assess the changes in pupils' hygiene behaviour or the impact on the hygiene practices and health of family members, this model of hygiene promotion is quite easy to replicate and should be useful for improving hygiene habits. It could potentially bring about a strong reduction in carriage of intestinal parasites. Furthermore, interventions that focus on hygiene have been associated with a reduction in the number of visits to health centres [24]. Programmes such as this, which promote better hygiene, should therefore be encouraged in low income countries.

Our assessment of the health impact of building latrines and implementing an educational programme confirms the findings of Corrales et al. on the fact that transmission of infections is not only linked to latrines. There appears to be some interaction of various socioeconomic and cultural factors [11].

## 5. Conclusion

Putting health infrastructure in place in schools obviously had an impact on hygiene-related habits in the beneficiary schools and communities. This impact is not generally demonstrated in health (parasitic diseases, and symptoms related to lack of hygiene). Such activities nevertheless have at least short term beneficial effects since they increase access to clean drinking water and teach children hygienic habits. This contributes to improving their health.

Monitoring should continue over a longer period, in order to consolidate the effects of such interventions. Awareness-raising on standard hygiene measures, especially hand washing and use of latrines, should be carried out regularly.

## Figures and Tables

**Table 1:** Timetable of activities: ESAMIS project and CERMES surveys.

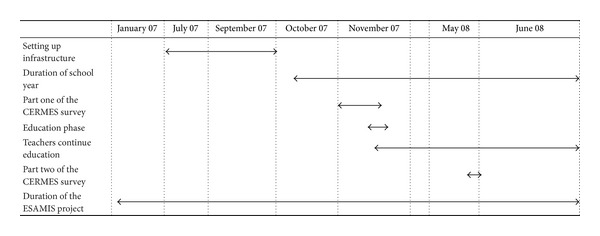

**Table 2:** Sex, frequency of functional signs due to lack of hygiene, absence from school, pupils' hand washing, and latrine use before and after the project, Maradi, Niger.

	Before the project		After the project		
	*n*	% per group	*n*	% per group	
Pupils selected					
Programme group	343	51.6	359	51.6	
Control group	322	48.4	337	48.4	
Total	**665**		**696**		

	Before the project		After the project		
	*n*	% of girls	*n*	% of girls	

Breakdown by sex					
Programme group	343	42.3	359	41.8	
Control group	322	28.6	337	31.8	
Total	**665**	**35.6**	**696**	**36.9**	

	Before the project		After the project		
	*n*	Prevalence (%)	*n*	Prevalence (%)	

Diarrhoea					
Programme group	343	3.2	356	3.1	
Control group	322	3.2	335	2.4	
Total	**665**	**3.9**	**691**	**2.7**	*P* = 0.04
Abdominal pains					
Programme group	343	5.2	356	3.1	
Control group	322	4.0	334	3.3	
Total	**665**	**4.7**	**690**	**3.2**	*P* = 0.02
Vomiting					
Programme group	343	2.0	356	1.1	
Control group	322	1.2	334	1.5	
Total	**665**	**1.7**	**690**	**1.3**	*P* = 0.08

	Before the project		After the project		
	*n*	Frequency (%)	*n*	Frequency (%)	

Absence from school					
Programme group	343	4.4	359	8.6	
Control group	322	0.6	337	5.9	
Total	**665**	**2.6**	**696**	**7.3**	*P* < 0.0001
Latrine use at home					
Programme group	330	24.8	359	29.9	
Control group	322	6.8	334	3.3	
Total	**652**	**16.2**	**693**	**17.1**	*P* = 0.20

**Table 3:** Percentage of use of drinking water sources at school before and after the project.

	Before the project				After the project			
	Total	Water tap	Borehole	Well	Total	Water tap	Borehole	Wells
Programme group	92	87.0	2.2	10.9	270	99.6	0.4	0.0
Control group	39	0.0	10.3	89.7	95	1.1	42.1	56.8
Total	**131**	**61.1**	**4.6**	**34.4**	**365**	**74.0**	**11.2**	**14.8**

**Table 4:** Comparison of intestinal parasite carriage rates (%) before and after the project.

	Before the project		After the project		*χ* ^2^	*P*
	Total	Prevalence (%)	Total	Prevalence (%)		
Programme group	343	9.6	359	8.1	0.34	0.55
Control group	321	5.3	337	12.5	9.6	0.001
Total	**664**	**7.5**	**696**	**10.2**	**2.7**	**0.10**

**Table 5:** Prevalence of parasites found before and after the project in programme schools and control schools.

Parasite	Before the project		After the project		*χ* ^2^	*P*
	Number positive	Prevalence (%)	Number positive	Prevalence (%)		
*Hookworm *						
Programme group	1	0.3	0	0	5*e* − 04	0.98
Control group	0	0	0	0		

*Ascaris lumbricoides *						
Programme group	0	0	0	0		
Control group	1	0.3	0	0		

*Pinworm *						
Programme group	3	0.9	8	2.2	1.3	0.25
Control group	2	0.6	11	3.3	4.63	0.03

*Entamoeba histolytica *						
Programme group	1	0.3	0	0	5*e* − 04	0.98
Control group	0	0	0	0		

*Trichuris trichiura *						
Programme group	3	0.9	0	0	1.43	0.23
Control group	0	0	0	0		

*Hymenolepis nana *						
Programme group	0	0	7	1.9	0.92	0.02
Control group	5	1.5	11	3.3	1.36	0.24
